# Immunohistochemical, functional, and anatomical evaluation of patients with idiopathic epiretinal membrane

**DOI:** 10.1007/s00417-023-06366-w

**Published:** 2024-01-10

**Authors:** Julio Cesar Molina Martín, Laura Fernández Sánchez, David P. Piñero, Nicolás Cuenca Navarro

**Affiliations:** 1 Department of Ophthalmology, San Juan University Hospital, N-332, S/NSant Joan d’Alacant, 03550 Alicante, Spain; 2 Department of Optics, Pharmacology and Anatomy, University of Alicante, Crta San Vicente del Raspeig S/NSan Vicente del Raspeig, 03690 Alicante, Spain; 3 Department of Physiology, Genetic and Microbiology, University of Alicante, San Vicente del Raspeig, Alicante, Spain

**Keywords:** Epiretinal membrane, Pars plana vitrectomy, Müller cells, Astrocytes, Microglia, Macrophages, Spectral-domain optical coherence tomography, Immunohistochemical analysis

## Abstract

**Purpose:**

The main purpose of this study was to perform an immunohistochemical, functional, and anatomical evaluation of patients with idiopathic epiretinal membrane (ERM).

**Methods:**

Twenty-four specimens of idiopathic ERM from 24 consecutive patients who underwent 23 G pars plana vitrectomy for ERM and internal limiting membrane (ILM) peeling at the San Juan University Hospital in Alicante (Spain) in 2019 were analyzed. All patients underwent a complete ophthalmological examination including measurement of best corrected visual acuity (BCVA) and macular analysis by spectral-domain optical coherence tomography (SD-OCT) at the time of diagnosis and 3 months after surgery. Specific glial fibrillar acid protein antibodies (GFAP) and S100 calcium-binding protein β (S100β) immunostaining markers were used to identify the macroglial component of the ERM, Müller cells, and astrocytes. Ionized calcium-binding adapter molecule 1 protein (Iba1) antibodies were used as specific markers for inflammatory cells, such as microglia and macrophages.

**Results:**

Mean preoperative BCVA measured with Snellen chart was 0.3 and 0.6 preoperatively and at 3 months after surgery, respectively. SD-OCT identified 15 patients (62.5%) with a disruption of the outer retinal hyperreflective bands. The immunohistochemical study showed the presence of Müller cells in almost all cases (91.6%), as well of abundant microglia and macrophages. Microglia and macrophages were more frequently present in earlier stages of ERM. Microglia were present in ERM independently of the outer retinal hyperreflective bands integrity as measured by SD-OCT. A greater presence of macrophages was found in those ERMs with no outer retinal hyperreflective band disruption.

**Conclusions:**

Müller cells seem to be the most frequent cell group in ERMs, with also presence of microglia cells and macrophages. Astrocytes were more frequently found in early stages of ERMs. Microglia and macrophages were most frequent in ERMs with early stage (1, 2, or 3) than in advanced stages (4).

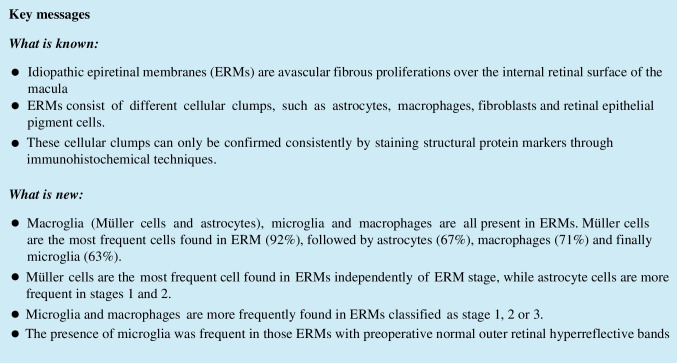

## Introduction

**Fig. 1:**
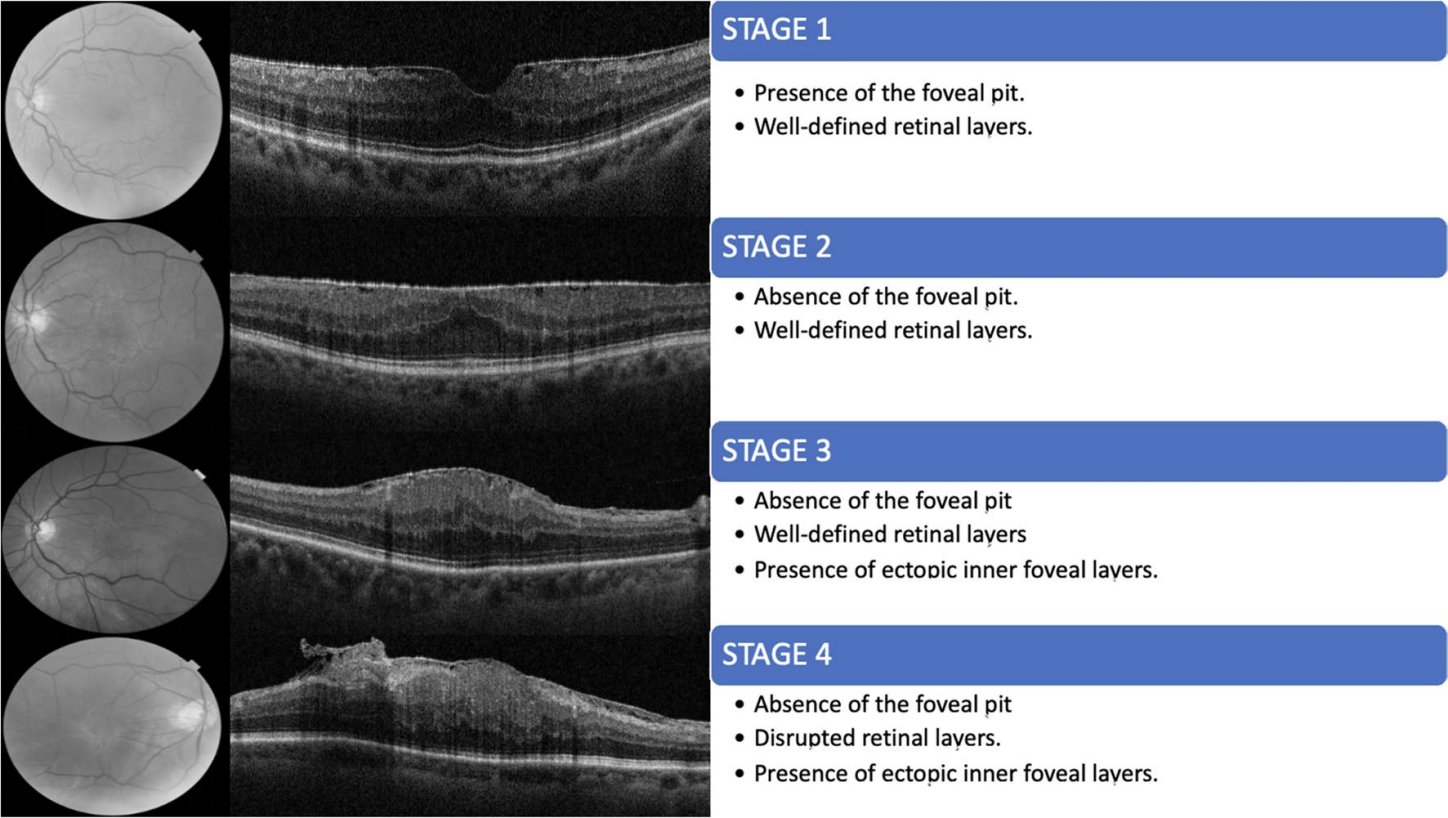
Retinal cross-section images obtained by means of SD-OCT in four cases from the sample evaluated before (left: A1, B1, C1, and D1) and 3 months after surgery (right: A2, B2, C2, and D2). The anatomical recovery of the retinal layers can be easily seen after surgery

Epiretinal membranes (ERMs) are avascular fibrous proliferations over the internal retinal surface of the macula, although in rare cases, a small vascular component may be present as a possible secondary transformation of the primary fibrocellular membranes. The presence of this tissue in the inner part of the macula has a very wide range of clinical features, from being asymptomatic to generating metamorphopsias [1]. In an attempt to achieve a better forecast for visual recovery, many classifications of the ERMs have been proposed, almost all of them based in morphological features observed in optical coherence tomography (OCT) [2–4], although other approximations based on gene expression profile have been proposed [5]. One of the most widely used classifications was made by Govetto et al. (Fig. 1) [3]. These authors defined four categories according to the presence of ectopic inner foveal layers (EIFL) as seen with OCT. This classification, although useful, seem to be not enough to provide a more adequate forecast. Recently, a new interpretation of OCT images pay attention on the most outer retinal hyperreflective bands (ORHB) [6]. The ORHB were defined by Cuenca et al. in 2017 [6]. Layer 1 corresponds to the external limiting membrane (ELM), layer 2 corresponds to the external part of the photoreceptor inner segment, layer 3 corresponds to the phagosomes present in the apical part of the RPE cells, whereas layer 4 corresponds to mitochondria present at the base of RPE cells [6]. ERMs can be divided into idiopathic, if they present primarily, or secondary if they are associated to an existing disease, such as a vascular disease, trauma, or intraocular inflammation.

In the formation of idiopathic ERM, an important role of the posterior vitreous detachment has been proposed. The vitreous detachment can trigger cell migration and ERM formation [7]. Gass et al. [2] suggested that ERMs consist of different cellular clumps, such as astrocytes, macrophages, fibroblasts, and retinal epithelial pigment cells (REP). Morphological identification by electronic microscopy of ERM cells can be challenging due to transdifferentiating of these cells [8]. Glial cells, fibroblasts, and RPE cells change their morphology when cultivated in vitreous medium and their ultrastructure is undistinguishable according to Vinores et al. [9] Therefore, staining structural protein markers through immunohistochemical techniques are needed in order to properly identify these cells.

The main purpose of this study was to perform an immunohistochemical, functional, and anatomical evaluation of patients with idiopathic ERM, investigating the potential presence of inflammatory cells in ERM specimens by immunohistochemical study and their possible relationship with the integrity of outer retinal hyperreflective bands (ORHB).

## Methods

### Clinical protocol

An observational study including 24 specimens of idiopathic ERM from 24 consecutive patients who underwent 23 G pars plana vitrectomy (PPV) for ERM and internal limiting membrane (ILM) peeling with intraoperative staining (Membrane Blue® Dual, DORC®) was conducted at the San Juan University Hospital in Alicante, Spain. Patients with other retinal pathologies were excluded from the study. The study was approved by the ethics committee of San Juan University Hospital of Alicante (Spain) and complied with the Declaration of Helsinki. All patients signed an informed consent prior to the inclusion in the study.

**Fig. 2:**
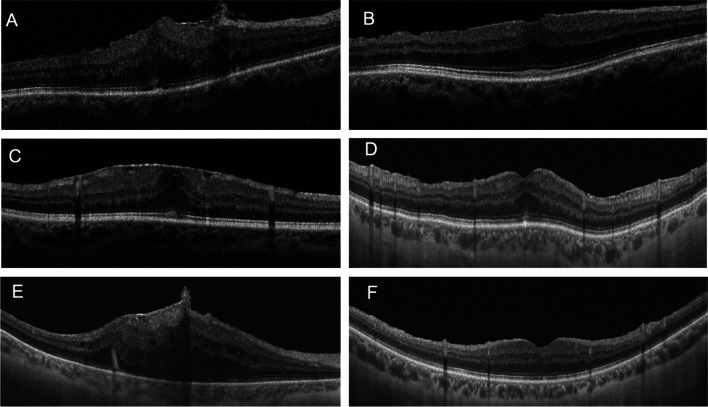
Integrity of the outer retinal hyperreflective bands (ORHB) before (**A**, **C**, **E**) and after (**B**, **D**, **F**) ERM surgery

Clinical examinations were performed before surgery and repeated at the first and third months after surgery. The examinations included measurement of best corrected visual acuity (BCVA) using Snellen charts, as well as comprehensive slit-lamp ophthalmologic and fundus examinations. ERMs were preoperatively confirmed by SD-OCT (TOPCON® 3D OCT-1 MAESTRO) using the 6 × 6 mm radial protocol centered in the fovea and only considering images with signals higher than 9/10. Two retinal specialists interpreted the OCT images (JM and JG). Idiopathic ERMs were classified according to the Govetto et al. classification (Fig. 1) [3]. All OCT images were analyzed and classified according the ORHB integrity (Fig. 2). ORHB was considered as intact when continuous and regular hyperreflective bands at the fovea could be detected. On the contrary, disruption of the ORHB was defined as an interruption, rupture, or disappearance of such bands.

### ERM specimen processing

All ERM specimens were obtained during the PPV surgeries and fixed in a 4% paraformaldehyde with a phosphate buffer (PB) (NaHPO_4_ and NaH_2_PO_4_, 0.1 M, pH 7.4) for an hour. Once fixed, specimens were washed out three times with the PB for 5 min in order to remove excess fixative. Afterwards, specimens were cryoprotected with increasing concentration of sucrose solutions: 15% (1 h), 20% (1 h), and 30% (overnight). After this, the samples were deep-frozen in isopentane and liquid nitrogen and kept at − 80 °C until used for posterior analysis.

### Antibodies used

Different specific cell biomarkers were used in order to identify and differentiate macroglia (Müller cells and astrocytes) and microglia. Table 1 summarizes all primary antibodies used. Specific glial fibrillar acid protein antibodies (GFAP) were used to identify the macroglial component (Müller and astrocytes) that is present in the ERMs. Antibodies against S100 acidic calcium binding protein β (S100β) were also used to identify Müller cells [10, 11]. Ionized calcium-binding adapter molecule 1 protein (Iba1) antibodies were used as specific markers for inflammatory cells, such as microglia and macrophages. In addition to the specific glial markers, the morphological features were also considered to identify each cell population. Secondary antibodies used were Alexa Fluor 488-conjugated anti-rabbit, Alexa Fluor 488-conjugated anti-mouse, Alexa Fluor 488-conjugated anti-goat, Alexa Fluor 555-conjugated anti-mouse, and Alexa Fluor 555-conjugated anti-rabbit made in donkey. All secondary antibodies used were purchased from Molecular Probes (Eugene, OR) and the working dilution was 1:100.

**Table 1:** Primary antibodies used for immunohistochemistry characterization of the ERM specimens

Molecular marker (initials)	Antibody (reference)	Source and catalog number	Working dilution
Glial fibrillary acidic protein (GFAP)	Mouse monoclonal, clone: G-A-5	Sigma (G 3893)	1:200
Glial fibrillary acidic protein (GFAP)	Rabbit polyclonal	Dako (N1506)	1:20
Ionized calcium-binding adapter molecule 1 (Iba-1)	Rabbit polyclonal	Wako Chemicals, Richmond, VA, USA (019–19741)	1:500
Collagen type IV	Goat polyclonal	Millipore (AB769)	1:200
S100 (b-subunit)	Mouse monoclonal, clone: SH-B1	Sigma-Aldrich (S2532)	1:500

### Immunohistochemical staining

ERMs were thawed right before their processing. Specimens were rinsed three times with a PB for 5 min to wash out any saccharose left over. Afterwards, in order to avoid any cross reaction with secondary antibodies, specimens were incubated in donkey’s serum with 10% PB supplemented with 1% Triton X-100 for 1 h in a wet chamber. Then after, the tissue was washed out three times in PB for 5 min. After serum blockage, sections were incubated for 24 h under mild agitation at room temperature with a combination of primary antibodies at an optimal concentration (Table 1).

After 24-h incubation, the tissue was washed out again with PB for 5 min in order to eliminate any primary antibody excess. After the rinse, ERMs were cultured with the corresponding secondary antibodies. Secondary antibodies were used at a 1:100 dilution, while the nuclear marker TO-PRO 3-iodide from Molecular Probes (Eugene, OR) was used at a 1:1000 dilution. Incubation time was 2 h in complete darkness and mild agitation. After that, specimens were washed out three times with PB and mounted with Citifluor mountant media (Citifluor Ltd, London, UK) and coverslipped for further observation.

A total of 24 immunohistochemistry marked ERMs were included for a qualitative analysis allowing the determination of the presence or absence of certain cellular clumps attending to GFAP and Iba1 markers and their morphological characteristics. The results obtained were statistically analyzed and correlated with SD-OCT images. Images were obtained with a confocal fluorescent microscope (Leica TCS SP2, Leica Microsystems, Wetzlar, Germany). Brightness and contrast of the images were adjusted with Adobe Photoshop CS2 (Adobe Systems, San Jose, CA).

### Statistical analysis

All analyses were conducted using the SPSS 15.0 software (IBM, Armonk, NY, USA). The normality of data distribution was assessed using the Kolmogorov–Smirnov test. Average, standard deviation, median, and range were calculated for the quantitative variables. The significance of the differences between preoperative and postoperative visual acuity was evaluated using the Wilcoxon test. *P* < 0.05 was considered as denoting statistical significance.

## Results

The baseline characteristics of the patients are shown in Table 2. Twenty-five ERMs were obtained and studied from 24 patients included in this study. Of the 24 patients, 16 were men and eight were women, ranging in age from 60 to 78 years (mean age, 69.3 years). An intact ORHB was observed on the SD-OCT images in nine patients (37.5%), while in the remaining 15 patients (62.5%), disruptions of ORHB were noted. Figure 2 shows the OCT profiles from four patients (A–D) in the preoperative exam (A1–D1) and at 3 months after surgery (A2–D2). The ERM can be easily observed in the preoperative OCT examination in all patients. In all cases, a clear recovery of the anatomical structure of the macula can be observed in the postoperative OCT images when compared to the preoperative images. No vascular component was observed in any of the membranes evaluated.

**Table 2:** Baseline characteristics of the sample evaluated

Number of patients	24
Number of eyes that underwent surgery	24
Male, *n* (%)	16 (66.7%)
Females, *n* (%)	8 (33.3%)
Mean age ± SD (range)	69.3 ± 8.1 (60–78)
Mean preoperative decimal BCVA ± SD	0.30 ± 0.13
Mean postoperative decimal BCVA ± SD	0.60 ± 0.15
ERMs stained with immunohistochemistry techniques, *n* (%)	24 (100%)
Presence of hyperreflective outer retinal bands disruption (preoperative) by SD-OCT, *n* (%)	15 (62.5%)
Absence of hyperreflective outer retinal band disruption (preoperative) by SD-OCT, *n* (%)	9 (37.5%)

*SD*, standard deviation

**Fig. 3:**
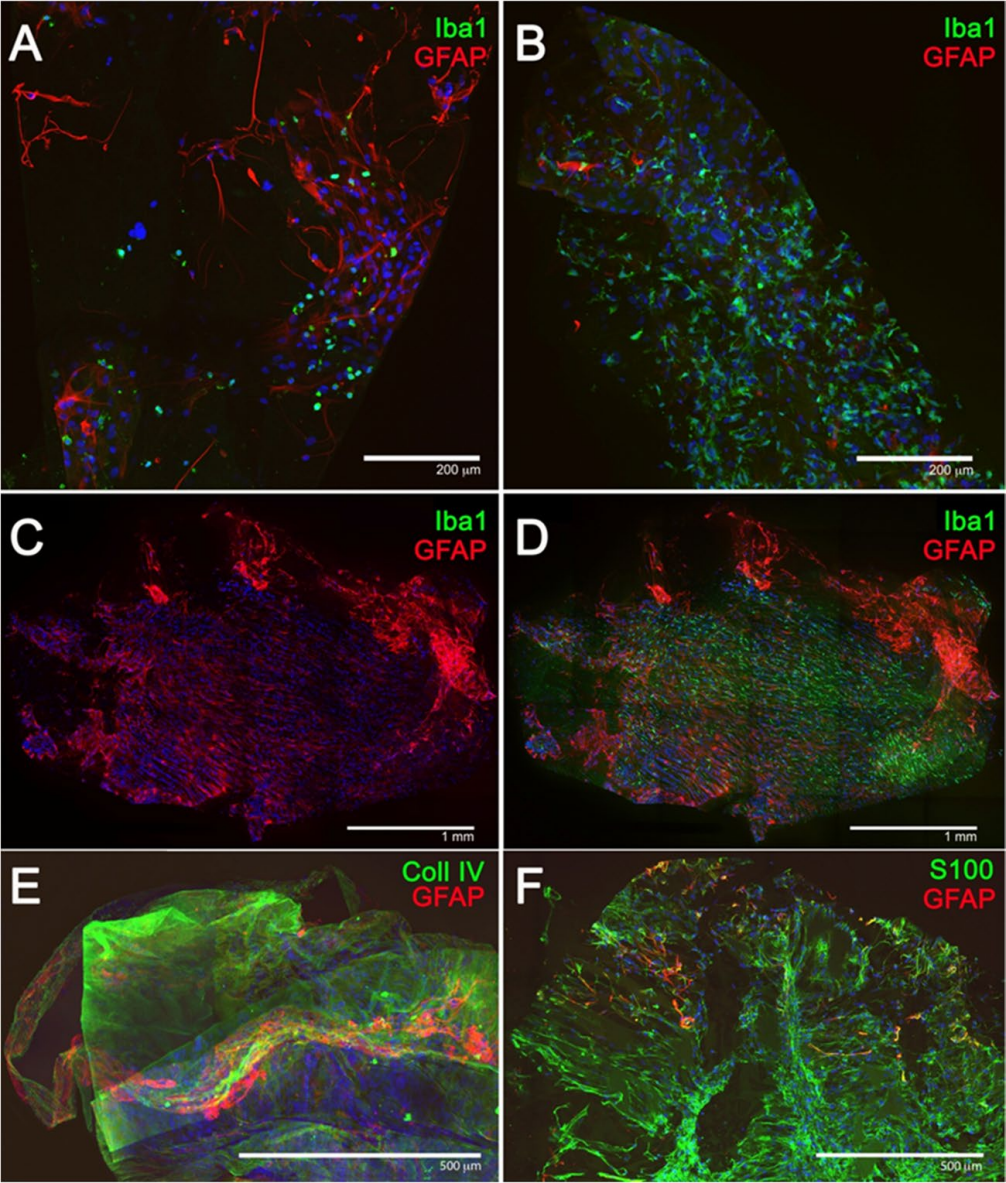
ERM specimens evaluated by staining with different immunomarkers: glial cells stained with GFAP antibodies (red) (**A**–**D**), microglia cells/macrophages stained with Iba-1 antibodies (green) (**A**–**D**), collagen IV protein stained with anti-collagen-IV (green) (**E**), and glial cell stained with anti-GFAP (red) and anti-S100b (green) proteins (**F**)

Figure 3 shows in a panoramic view, the number and distribution of the cells that could be found in the analyzed ERM. The immunostaining with antibodies against GFAP (red) and Iba-1 (green), both markers of macroglia and microglia/macrophages cells, respectively, showed clear differences between the membranes based on the number and distribution of these cell populations (GFAP + and Iba1 +). ERM with sparse Iba1 cells were found (Fig. 3A), as well as others ERM with higher density of Iba1 cells and low presence of GFAP cells (Fig. 3B) and ERMs with high density of both cell types (Fig. 3C). The presence of acellular component in the ERM was confirmed by immunolabelling with antibodies against collagen IV (Fig. 3C, green), as previously described by Bu et al. [12] The labelling of glial cells with two markers, GFAP and S100β (Fig. 3F), revealed two well-differentiated populations: a population of GFAP + /S100β + cells and another population of GFAP − /S100β + cells.

**Fig. 4:**
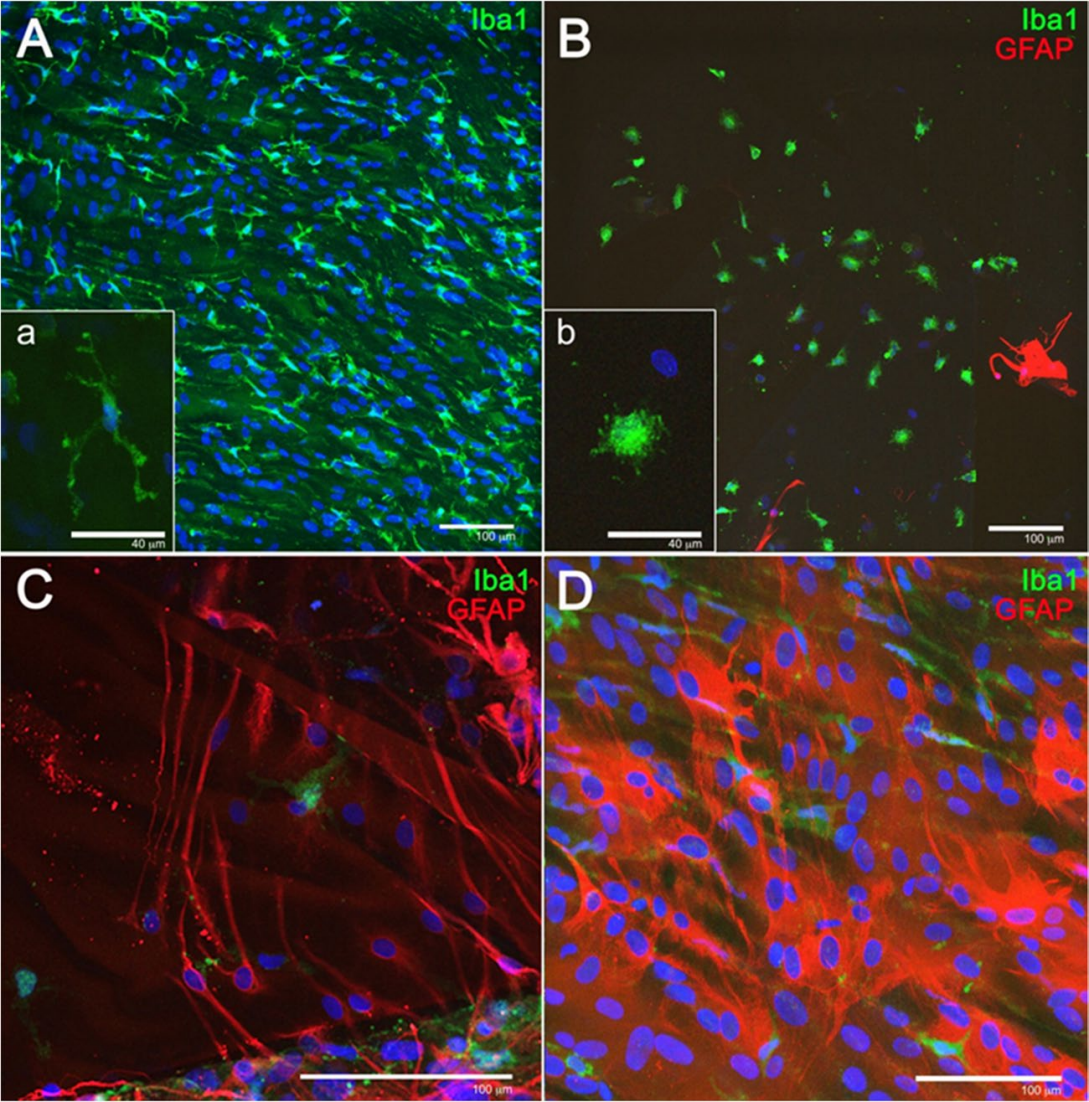
Morphological details of cells present in the ERMs evaluated: glial cells and microglia/macrophages stained with anti-GFAP (red) and anti-Iba1 (green) antibodies (**A**, **B**), and cells with a similar morphology to Müller cells (**C**) and astrocytes (**D**) with GFAP staining

In order to better understand the cell populations that form the ERM, a detailed study of the morphology of different cells immunolabelled against GFAP and Iba1 was performed. Figure 4 shows the morphological details of cells that were present in the specimens analyzed. First, labelling with Iba1 revealed cells with morphological characteristics of microglia (Fig. 4A), showing small nuclei and thin, long, and branched processes. Moreover, Iba1 + cells with amoeboid morphologies that unequivocally resemble macrophages were also observed (Fig. 4B). Furthermore, GFAP labelling revealed two basic types of morphology, some cells with spindle-shaped morphology, reminiscent of the morphology of Müller cells (Fig. 4C), and other cells with a stellate morphology, with numerous extensions of variable length and large nuclei, cells with a high similarity to retinal astrocytes (Fig. 4D).

**Fig. 5:**
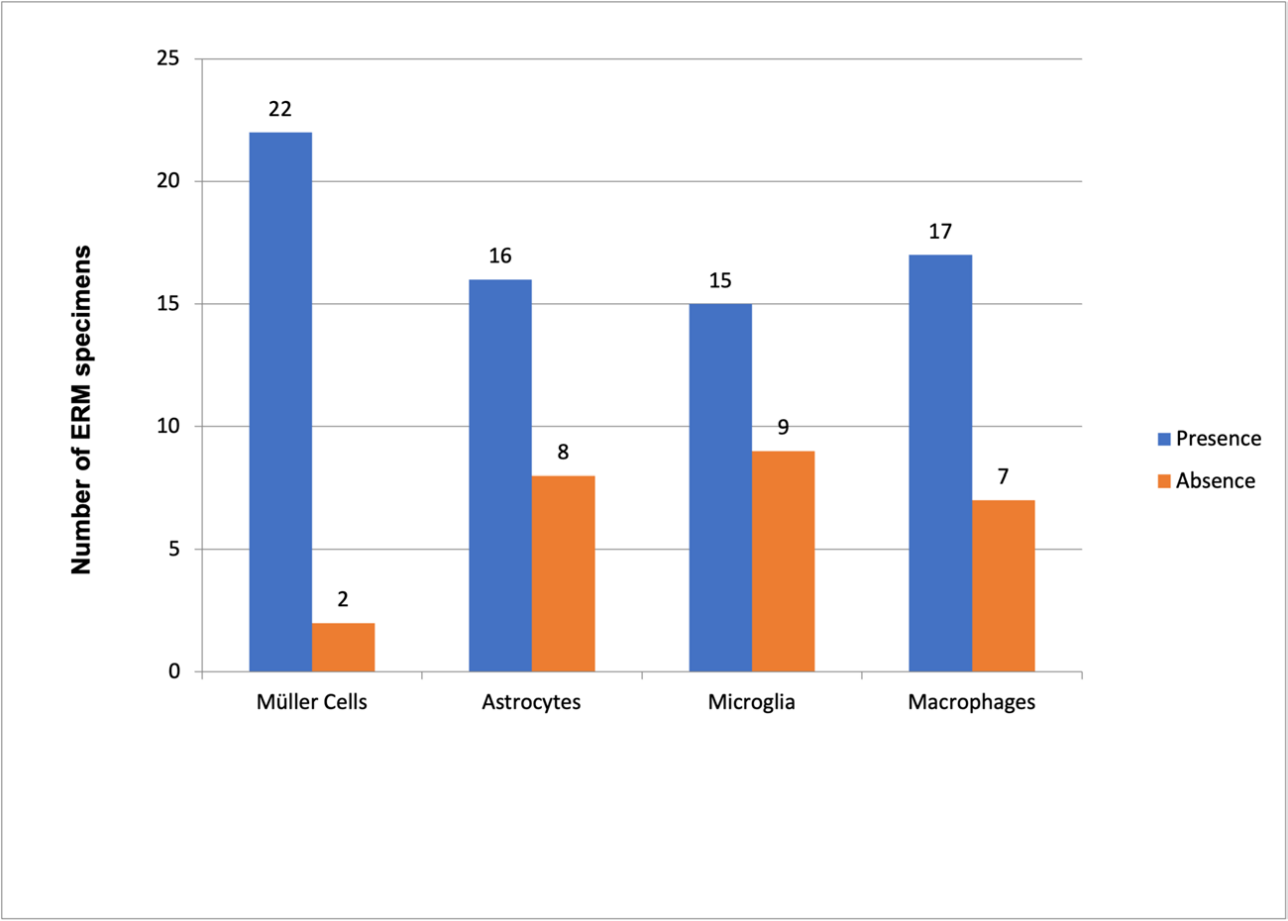
Graphical representation of the different cell types found in the epiretinal membranes (ERMs) evaluated. Glial cells, mainly Müller cells, were present in almost all ERMs, while astrocytes were identified in the 64% of the ERMs. Macrophages were more frequently found (68%) than microglia (36%) in ERMs

Figure 5 shows the distribution of the type of cells found in the ERMs evaluated. As shown, Müller cells identified with GFAP staining and morphological features were present in the 91.7% (22 of the 24) of the ERMs analyzed while astrocytes were present in the 66.7% (16 of the 24). Immune cells were identified as Iba1 + cells and the classification into microglia or macrophages was made following morphological features. As shown in Fig. 5, iba1 + cells with microglia-like shape were identified in 62.5% (15 of the 24) ERMs, while macrophages were found in 70.8% of the ERMs analyzed (17 of the 24).

**Fig. 6:**
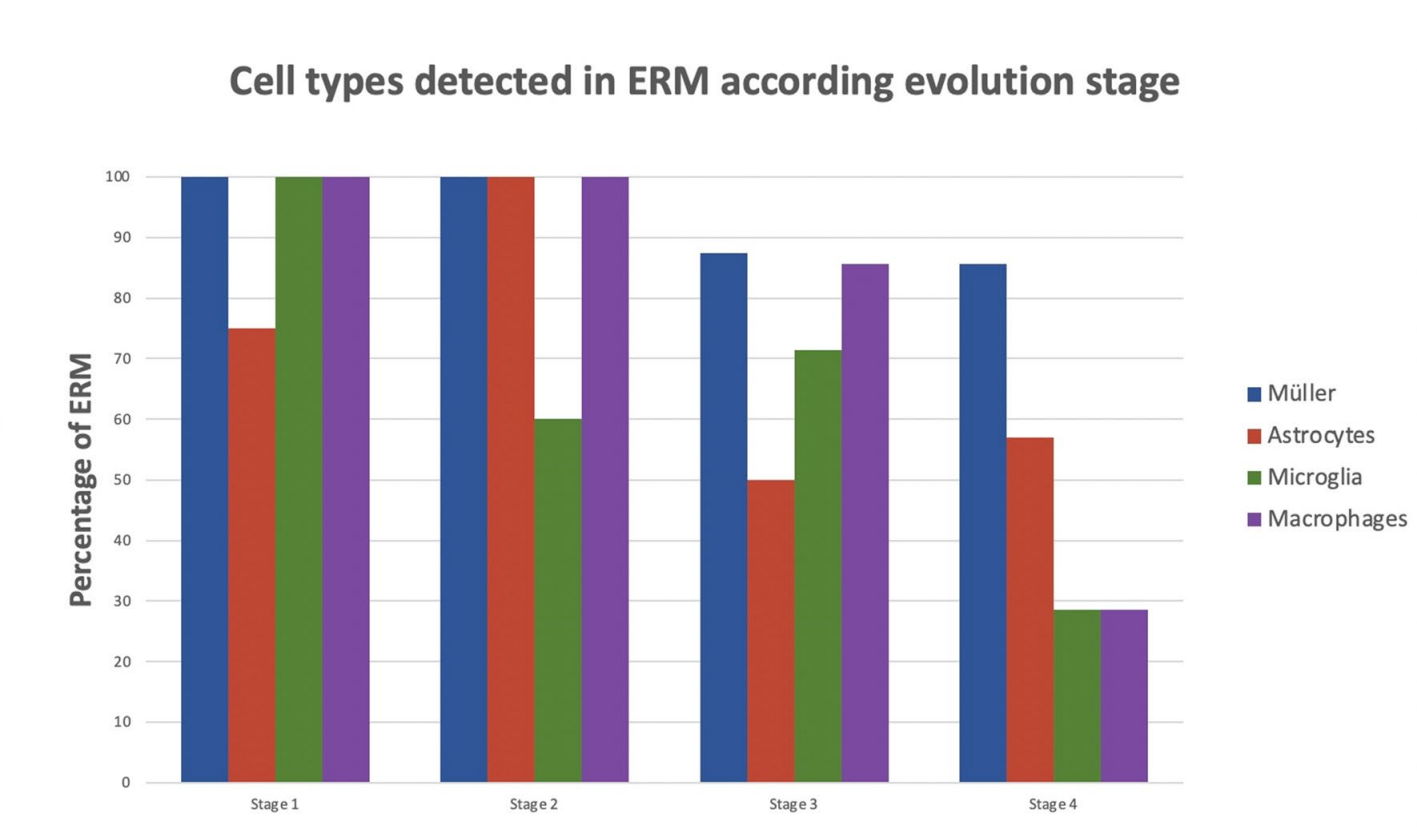
Cell types detected in the samples according to ERM stages (according to Govetto’s classification. [3]). Müller cells were the most frequent cell found in ERMs. Astrocytes were more frequent in the stages 1 and 2. Microglia was detected more frequently in stage 1 than in the rest while macrophages were most frequent in stages 1, 2, and 3

All membranes were classified according Govetto’s classification [3], of the 24 membranes included in this study, four were on stage 1, five on stage 2, eight on stage 3, and seven on stage 4. The distribution of the cells according to the membrane stage (Fig. 6) revealed that Müller cells were detected in the 100% of membranes in stages 1 and 2 and above 85% of the membranes in stages 3 and 4, being the most frequently cells found in membranes, independently of the membrane stage. Astrocytes cells were more frequent in membranes on stages 1 and 2 (75 and 100%, respectively) than in membranes on stage 3 or 4 (50% and 57.1% respectively). Microglial cells were more present in membranes in stage 1 of evolution (100%), its presence decreased slightly in stages 2 and 3 (60% and 71.4% respectively), while these cells were detected only in 28.6% of the ERMs in stage 4. Finally, macrophages were present in all the membranes in stages 1 and 2 (100%). The presence of macrophages in stage 3 was detected in 85.7% of the ERMs analyzed, while they were only present in the 28.6% of the membranes classified as stage 4. Therefore, it can be concluded that Müller cells were found in almost all membranes while astrocytes were found more frequently in stages 1 and 2. Microglia and macrophages were more frequent in early stages (stages 1, 2, and 3, Govetto classification [3]).

**Fig. 7:**
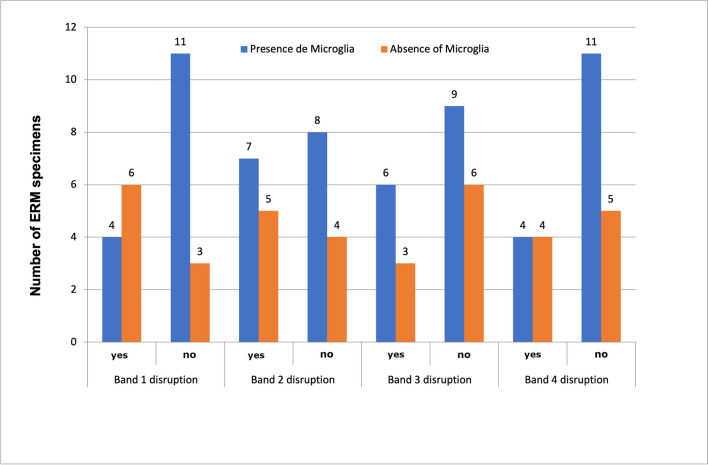
Presence of microglia according to outer retinal hyperreflective band disruption

**Fig. 8:**
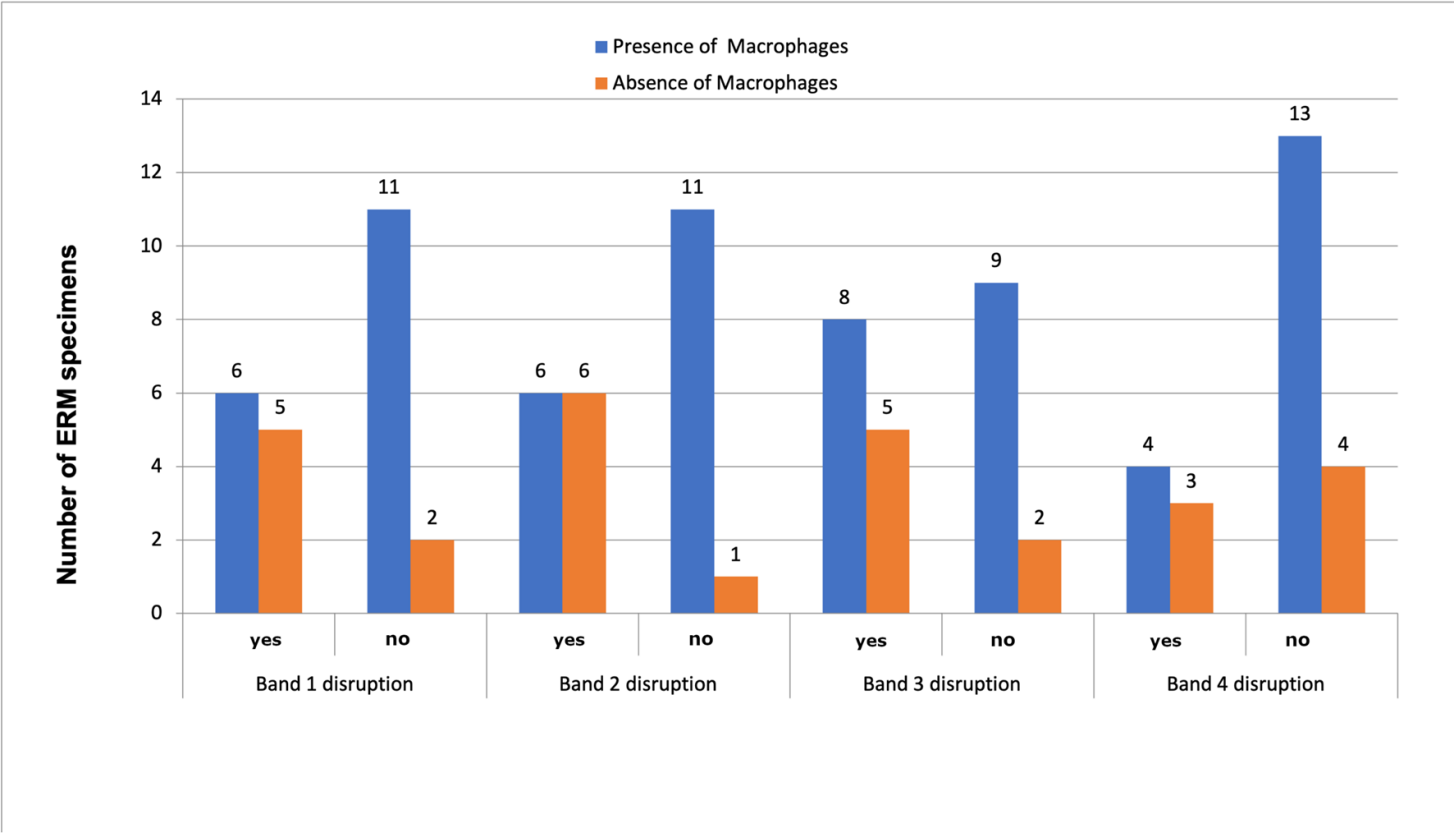
Presence of macrophages according to the outer retinal hyperreflective bands disruption. Macrophages were more frequent in those ERMs with no hyperreflective band disruption

Concerning the presence of microglia in ERMs as a function of the hyperreflective outer bands integrity measured by SD-OCT, it was independent from the absence or presence of disruption of any of the four hyperreflective outer bands (Fig. 7). In addition, a greater presence of macrophages was found in those ERMs without outer retinal hyperreflective bands disruption (Fig. 8).

## Discussion

In the sample evaluated in the current study, the mean BCVA using a Snellen chart at baseline and at 3 months after the surgery was 0.3 and 0.6, respectively (Table 2). The postoperative BCVA values were significantly better than those found at the baseline (*p* < 0.001). Similar results have been reported by Dawson and colleagues, [13] who carried out a prospective study from 2001 to 2011, evaluating 237 ERMs operated patients through PPV. There are some predictive factors for BCVA improvement after ERM surgical removal including shorter duration of symptoms before surgery, lesser central foveal thickness at baseline identified by the OCT, good integrity of the inter segment/outer segment photoreceptor junction at baseline, and thinner ganglion cell inner plexiform layer at baseline [14].

When performing the immunohistochemical study of the ERM samples obtained during surgery, macroglia (Müller cells and astrocytes), microglia, and macrophages were all present in ERM specimens. Macroglia cells were the most frequent cell type found, with a predominance of Müller cells (91.7% of specimens) versus astrocytes (66.7%). An interesting finding was the presence of complete Müller cells, including its nucleus and not only its cellular projections (Fig. 4C). Macroglia, and specially Müller cells, are relevant in idiopathic ERM formation since they are believed to be responsible for migrating towards the inner surface of the retina and develop an ERM. It is generally accepted that macroglial cells conform the ERM. However, there is a disagreement concerning which macroglial cells, Müller cells or astrocytes, are the main type of cell involved. According to Foos et al., [15] it is unlikely that ERMs derive from Müller cells since these are anchored in the outer retina, linked to the photoreceptors. On the other hand, Kase et al. [16] stated that Müller cells and its processes were the main constituents of idiopathic ERMs. Other studies [17] have suggested that there is a wide variety of cells, hyalocytes, RPE cells, fibroblasts, and microfibroblasts that account for ERMs. However, since cells in the vitreous may undergo important morphological changes through transdifferentiating, morphological criteria are inadequate to identify the origin of the cells involved [18]. For this reason, it is currently necessary to identify these cells through immunohistochemistry staining techniques, identifying structural proteins such as the glial fibrillar protein.

In this study, the immunohistochemical analysis has not only identified Müller cells and astrocytes, but also other cell types such as microglia and macrophages. Microglia derives from bone marrow blast cells that enter the central nervous system through vascularity during the embryogenesis [19]. These round cells are called ameboid cells and reside in the inner retinal layers, with short (activated) or long (resident) processes. In normal retinas, microglia is usually present around vascular inner surfaces and act as macrophages [20, 21]. An experimental study done on rats has shown that microglia activation triggered an increase in ameboid cells (activated microglia) that migrated towards the external layers of the retina and caused a liberation of microglia inhibitory factors that avoid photoreceptor cell death [22]. Provis et al. [23] described how certain microglial cells share some characteristics with presenting antigen dendritic cells. Therefore, microglia have the ability to present antigens and phagocyte waste material. According to this, the activation of microglia plays an essential role in retinal defense mechanisms against destructive environmental threats and in the facilitation of regeneration processes. Vishwakarma et al. [24] found activated microglia and oxidative stress and inflammation gene expression in ERMs removed from eyes with retinal detachment and proliferative diabetic retinopathy.

The current study describes the presence of different cell types (macroglia, microglia, and macrophages) in relation to the ERM staging as described by Govetto et al. [3] This classification is mostly based on the presence of ectopic inner retinal layers (EIFL) in SD-OCT. Müller cells and astrocytes were the dominant cell type in all ERM stages. However, other cell types, such as macrophages and microglia, were also prevalent during the initial stages. More advanced ERM stages showed a greater destructuring of internal and external layers, leading to a greater level of fibrosis. As we move towards more advanced stages with greater fibrosis and destructuring, there are less of these cell types. Coltrini and collleagues [5] found two differentiated clusters of ERM based on its gene profile expression, a cluster with less activity that they called transcriptionally quiescent and other cluster transcriptionally activated that could be related with the differences that we have found in ERMs from early and advanced stages. This should be investigated further in future studies.

The presence and active proliferation of immune cells in all idiopathic and secondary ERMs were demonstrated by Oberstein et al. [25] It is believed that there is a greater cell replication during ERM early stages compared to older ERMs. However, Oberstein et al. [25] showed that during the active vitreoretinal proliferation phase of the ERM, there is a lesser proliferation rate than expected. According to this, it can be hypothesized that most of the cell proliferation activity is a very early event that could take place before the actual formation of the ERM.

Concerning the relationship between immunohistochemical findings and outer retinal hyperreflective bands integrity, this study has tried to relate the presence of microglia and alterations in the ORHB measured by SD-OCT. Surprisingly, the presence of microglia was frequent in those ERMs with preoperative normal ORHB (Fig. 7).

This study has some limitations that should be acknowledged. First, the number of patients with ERM investigated and the number of ERM samples analyzed can be considered as limited, but they were enough to define clear preliminary histological findings to investigate further in future studies. Likewise, the inclusion of more cases from different stages would have allowed performing a more comprehensive characterization of ERMs and possibly to extract more conclusions about the physiopathological process of this condition.

In conclusion, Müller cells seems to be the most frequent cell group in ERMs, with also presence of microglia cells and macrophages. Astrocytes are more frequently found in ERMs on stages 1 and 2. Microglia and macrophages were more frequent in ERMs in the stages 1, 2, and 3 than in the 4 stage.
